# Antibody Response to a Live-Modified Virus Vaccine against Bovine Viral Diarrhoea in Dairy Cattle in a Field Trial

**DOI:** 10.3390/vaccines9030259

**Published:** 2021-03-15

**Authors:** Małgorzata D. Klimowicz-Bodys, Katarzyna Płoneczka-Janeczko, Michał Czopowicz, Mirosław Paweł Polak, Agnieszka Lachowicz-Wolak, Krzysztof Rypuła

**Affiliations:** 1 Division of Infectious Diseases of Animals and Veterinary Administration, Department of Epizootiology and Clinic of Birds and Exotic Animals, Faculty of Veterinary Medicine, Wroclaw University of Environmental and Life Sciences, pl. Grunwaldzki 45, 50-366 Wroclaw, Poland; malgorzata.klimowicz-bodys@upwr.edu.pl (M.D.K.-B.); katarzyna.ploneczka-janeczko@upwr.edu.pl (K.P.-J.); agnieszka.lachowicz-wolak@upwr.edu.pl (A.L.-W.); 2 Division of Veterinary Epidemiology and Economics, Institute of Veterinary Medicine, Warsaw University of Life Sciences–SGGW, ul. Nowoursynowska 159c, 02-776 Warsaw, Poland; mczopowicz@gmail.com; 3 Department of Virology, National Veterinary Research Institute, al. Partyzantów 57, 24-100 Puławy, Poland; ppolak@piwet.pulawy.pl

**Keywords:** BVDV, dairy cattle, life-modified virus vaccine, vaccination

## Abstract

(1) Background: The objective of the study was to evaluate the long-term antibody response of dairy cows to a single dose of a commercial modified-live virus (MLV) vaccine against bovine viral diarrhea (Mucosiffa^®^ CEVA Sante Animale, Liburne, France). (2) Methods: The study was carried out in a dairy cattle herd counting 290 animals negative for bovine viral diarrhoea virus (BVDV). The vaccination was implemented following the manufacturer’s instructions. Twelve dairy cows were randomly selected before the study, and blood samples were collected right before the vaccination and then 12 times at 1-month intervals. The serum samples were screened using a virus neutralization test (VNT) and ELISA. (3) Results: Both tests showed that antibody titers increased significantly in all animals within the first month post-vaccination, and continued to increase significantly until the second (VNT) and third (ELISA) month post-vaccination. Antibody titers remained high and stable until the end of the study. Moreover, cows did not show any adverse reactions or clinical symptoms of the disease. (4) Conclusion: The results of this study indicated that the administration of one dose MLV vaccine was able to stimulate long-lasting (12-months) and strong antibody response in all vaccinated cows.

## 1. Introduction

Bovine viral diarrhea virus 1 (BVDV-1) and bovine viral diarrhea virus 2 (BVDV-2), (according to the new taxonomic classification referred to as Pestivitus A and Pestivius B, respectively) are small single-stranded positive-sense RNA viruses, classified in the *Pestivirus* genus and the Flaviviridae family [1]. These viruses cause an infectious and contagious disease in cattle and wild ruminants, bovine viral diarrhea (BVD), characterized by various clinical signs relating to the gastrointestinal and respiratory systems. Viral infection induces immunosuppression and reproductive disorders as well. BVD causes large economic losses due to forced culling, morbidity and mortality [2,3]. The impact of BVDV depends on its virulence, the time of infection, herd immunity, level of disease prevalence, herd production level and concomitant infections [4,5].

BVDV-1 is endemic in many regions of the world including Poland [6,7]. BVDV infection has been found in 30–70% of cattle dairy herds [8,9] with a high percentage of BVDV-positive animals, especially in large dairy herds [10] in Poland. The vaccination with a modified-live virus (MLV) is currently a well-accepted procedure in the BVD control programs in most of the European countries and is considered a complementary biosecurity tool in countries with high BVD prevalence, in order to prevent infection and re-infections in cattle herds [11].

The vaccination has provided protection against BVDV that has been effective from a herd perspective, reduced clinical signs of disease and economic losses [4,5] although vaccines could have limited durations of immunity [12]. The value of vaccination is that it increases herd immunity so that the incidence of BVDV infection and the percentage of calves born as persistently infected (PI) are reduced in the herd as a whole [13]. For many years, the vaccination with MLV vaccines has been limited in pregnant cows [14], but now several MLV vaccines against BVD are approved for use during pregnancy and lactation, one of them being Mucosiffa^®^ (CEVA Sante Animale, Liburne, France). However, detailed data on the long-term antibody response to a single vaccination with a commercial MLV vaccine are scarce.

This study aimed to evaluate the long-term antibody response of dairy cows to a single dose of a commercial modified-life virus (MLV) vaccine against BVD.

## 2. Materials and Methods

### 2.1. Animals

The study was carried out on a dairy farm in Greater Poland voivodeship, between February 2018 and May 2019. The herd counted 290 heads. Infections with rotavirus, coronavirus and *Cryptosporidium parvum* in calves and *Streptococcus uberis* in adult cows had been previously detected in the herd. All animals were free from bovine herpesvirus type 1 (BHV-1). This herd had been involved in a voluntary BVD control program and had all of their animals previously tested for BVDV. All animals were analyzed with two commercial ELISAs: for BVDV antibody detection—IDEXX BVDV Total Ab Test (Scandinavia AB, Sweden) and for BVDV antigen detection—IDEXX BVDV Ag/Serum Plus ( Switzerland GmbH, Switzerland). The samples were tested individually. All tests were performed at the Diagnostic Laboratory EPI-VET of the Faculty of Veterinary Medicine, Wroclaw, Poland. The cattle were inseminated within 4–6 weeks after vaccination. Cows and their offspring were monitored after vaccination. The study was field research. No animals were introduced into the herd during the study.

### 2.2. Vaccination

The monovalent commercial vaccine Mucosiffa^®^ (CEVA Sante Animale, Liburne, France) containing live-attenuated, cytopathic BVDV C24V strain, subgenotype 1a at a minimum titer of 10^3.5^ TCID_50_ and maximum titer of 10^6^ TCID_50_ per 2 ml dose was used. The vaccination was started in March 2018. All animals were vaccinated following the manufacturer’s instructions.

### 2.3. Samples Collection

Twelve adult cows (1st, 2nd and 3rd lactation) from this herd were randomly selected for the study using a simple random method. This sample size was chosen to ensure at least 95% certainty (level of confidence) that at least one seronegative cow would have been detected if less than 75% of the vaccinated cows had seroconverted after vaccination. The following formula was used for the calculation of the sample size [15]:

(1) $$n=\left(1-{\left(1-P\right)}^{1/d}\right)\times \left(N-\frac{d}{2}\right)+1$$

where:

n—required sample size
N—herd size (290)
d—minimum expected number of animals that did not seroconvert after vaccination (25%)
P—the probability of finding at least one seronegative animal in the sample (i.e., a level of confidence = 95%)

Blood samples were taken from the jugular vein right before the vaccination (day 0) and then 12 times at one-month intervals. Blood was left at room temperature for 8–12 h after collection and centrifuged. Then, serum samples were frozen at −80 °C and transported directly to the Diagnostic Laboratory of Department of Virology of National Veterinary Research Institute, Pulawy, Poland, for virus neutralization test (VNT) and ELISA.

### 2.4. ELISA

Serum samples were tested with Erns Ab ELISA (BVDV Total Ab Test, IDEXX, Switzerland). Samples with sample-to-positive control ratio (S/P%) values higher than or equal to 30% were classified as positive, those with values less than 20% were considered negative and samples with values ranging from 20% to 29% were considered inconclusive. ELISA was performed according to the manufacturer’s manual at an absorbance of 450 nm wavelength. The test has been shown to be 96.7% sensitive and 97.1% specific compared with VNT [16].

### 2.5. Virus Neutralization Test (VNT)

VNT was done in two-fold serial dilutions (from 1:5 to 1:640) of serum samples from all the cows. All serum samples were tested for neutralizing antibodies against cytopathic (cp) BVDV-1a strain Singer used at the concentration of 100 TCID_50_. Viral controls included 100, 10, 1 and 0.1 TCID_50_. The cell culture control was Madin–Darby Bovine Kidney (MDBK) without BVDV infection. The antibody titer was read after 4 days of incubation at 37 °C and 5% CO_2_ atmosphere and expressed as the reciprocal of the highest dilution inhibiting cytopathic effect in 50% of dilution wells. The titer was presented as a binary logarithm (log_2_) value.

### 2.6. Statistical Analysis

Antibody titers were presented as the median, interquartile range (IQR) and range, and compared between consecutive months using the Friedman test and Wilcoxon signed-rank test with Bonferroni correction for familywise error. Correlation between VNT titers and ELISA S/P% was determined using Spearman’s rank correlation coefficient (R_s_). A significance level (α) was set at 0.05. Statistical analysis was performed in TIBCO Statistica 13.3.0 (TIBCO Software Inc., Palo Alto, CA, USA).

## 3. Results

At the beginning of the study, all animals were seronegative for antibodies against BVDV and negative for BVDV antigen. All newborn calves subsequently tested (as part of the BVDV monitoring program) were negative. 

**Figure 1:**
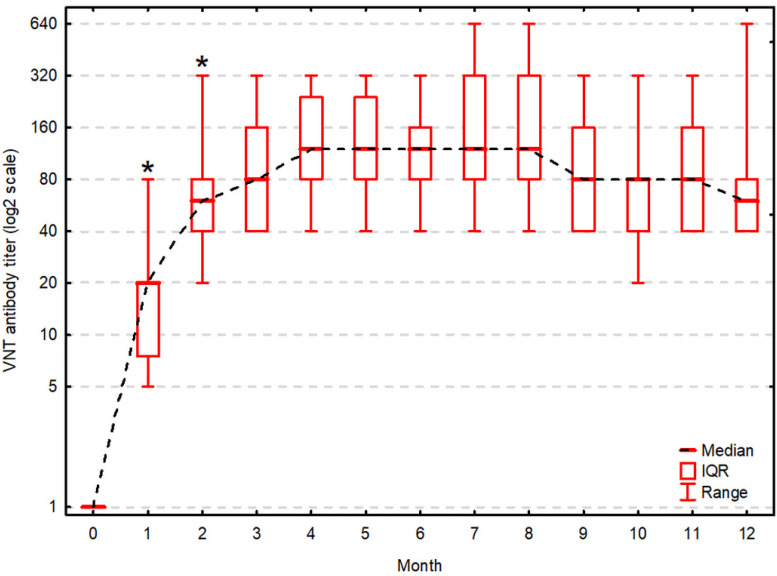
Antibody titers in virus neutralization test during 12 months post-vaccination. Antibody titers (on log base 2 transformed scale) are presented as the median, interquartile range (IQR) and range. Asterisk (*) indicates a statistically significant increase of the titer compared to the previous one (α = 0.05).

**Figure 2:**
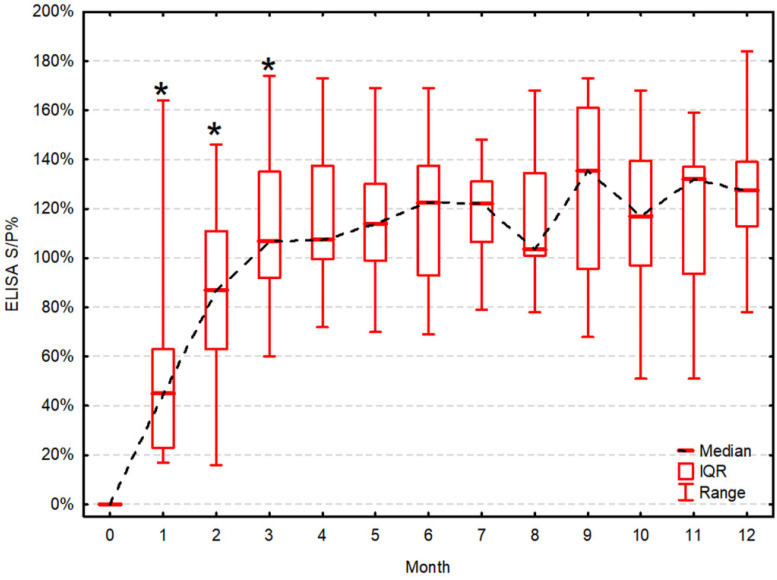
Sample-to-positive control ratio (S/P%) in ELISA during 12 months post-vaccination. S/P% in ELISA are presented as the median, IQR and range. Asterisk (*) indicates a statistically significant increase of the titer compared to the previous one (α = 0.05).

All cows showed a serological response to vaccination in both VNT and ELISA one month after administration of one dose of the live-modified vaccine. Then, antibody titers tended to increase significantly compared to the previous measurement on the 2nd month in VNT (Figure 1) and the 2nd and 3rd month in ELISA (Figure 2). The antibody titers remained high and stable until the end of the study. VNT titers were significantly positively correlated to ELISA results at all time-points except for the 9th month (Table 1).

Animals remained healthy throughout the study and no vaccinated cows aborted. No adverse reactions were observed following vaccination and no clinical signs of BVD disease were noted.

## 4. Discussion

The basis of successful BVDV eradication and control are: eradication efforts (identification and removing of PI animals), biosecurity and husbandry procedures to prevent the infections from being reintroduced as well as vaccination, or a combination of these factors. One of the main goals of vaccination against BVDV is to prevent persistent infection [17].

The aim of this study was to investigate the effectiveness of the live-attenuated (MLV) monovalent vaccine against BVDV, licensed for sale in Poland. Our study showed that vaccinated animals responded serologically to vaccination and had detectable VNT antibody titers to BVDV for at least one year following vaccination. Moreover, one dose of the commercial MLV vaccine ensures a protective level of antibodies in all vaccinated animals, which emerges as soon as one month after vaccination and remains stable for at least one year.

In our study, neutralizing antibody response was detected in vaccinated animals one month after vaccination, reaching a peak of 4.3 log_2_ titers. The antibody titer was significantly higher (5.8 log_2_, *p* < 0.05) a month later. Our results agree with those of Meyer et al. [18] who reported a similarly strong and durable neutralizing antibody response two months after Mucosiffa^®^ (CEVA Sante Animale, Liburne, France) vaccine administration. We also observed an increase of antibody titers after two months for VNT, although they were not statistically significant.

The VNT titer ≥ 1/20 (4.32 log_2_) which is considered as protective [19] was reached in all tested cows 2 months after vaccination and was maintained for the entire study period. The results of VNT were strongly positively correlated with the results of ELISA, which indicates that both tests yield consistent results.

Exposure of naïve cattle to the virus near the time of insemination could result in a reduction of pregnancy rates due to decreased conception rates and early embryonic death [17]. However, in our study, no reproduction disorders were observed. The calves were born healthy and negative for the BVDV antigen. Moreover, several commercial MLV BVDV vaccines have been validated for fetal protection when females were immunized prior to breeding [18,20]. It has been proven that a 3–6 weeks interval between vaccination and breeding or insemination is safe for both mothers and developing fetuses. It is probably safe to give a second dose of the MLV BVDV vaccination during the second half of gestation. However, there is a risk of fetal infection by the vaccine virus [21]. In our study, we used only one dose of vaccine 4 weeks before insemination and all tested animals responded positively to vaccination, so there was no need for a second vaccination. Griebel [21] concluded that there was no need for a booster vaccination during pregnancy which minimizes potential risk to the fetus and reduces cost for the producer.

Although vaccination does not always induce measurable antibodies in all individuals, BVDV vaccination increases herd immunity, so that the incidence of clinical disease and the percentage of PI calves born are reduced. The number of cows that were enrolled in our study provides very trustworthy information that at least three-fourths of cows in this herd mounted a similar serological response to the vaccination. Moreover, if exposure occurs, vaccination will also reduce the spread of the BVD virus in the herd [13]. Independently from the vaccination, regular monitoring of the farms should be undertaken as part of the BVDV control program.

The ability of MLV vaccines to induce an adequate neutralizing antibody titer is mainly associated with a reduction of viremia and viral shedding. However, the use of live-attenuated vaccines has been limited due to the use of BVDV contaminated fetal bovine sera (FBS). These live vaccines have the potential to increase the transmission of the virus and cause disease outbreaks in susceptible animals [13]. Raw FBS testing for the presence of BVDV before Gamma irradiation is demanded by most biopharmaceutical companies, which is required by the EU regulation Directive for Pharmaceutical Raw Material of Bovine Origin (EMEA-CPMP-BWP-1793-02). However, some FBS producers who want to avoid the risk that their products will not be approved for use in the production of bioproducts irradiate raw FBS prior to testing for BVDV, without disclosing this information to the biopharmaceutical companies [22]. Antos et al. [23] examined commercial FBS and vaccines available on the Polish market for immunprophylaxis and diagnostics. Only one serum showed the presence of an infectious virus and it was contaminated with two species of BVDV. Additionally, one inactivated vaccine against infectious bovine rhinotracheitis (IBR) was contaminated with BVDV-1.

## 5. Conclusions

BVDV vaccination increases herd immunity so that the risk of the incidence of animal acute disease and the percentage of PI calves are reduced. Given these promising results, we consider that the use of an MLV Mucosiffa^®^ (CEVA Sante Animale, Liburne, France) vaccine in a combination with a comprehensive BVDV control program should reduce the risk of BVDV infections in cattle herds.

## Figures and Tables

**Table 1:** Antibody titers in virus neutralization test (VNT) and sample-to-positive control ratio in ELISA during 12 months post-vaccination.

Test (Month)	VNT [log_2_ Transformed Titer]	Significance of an Increment Compared to the Previous Testing (*p*)	ELISA [S/P%]	Significance of an Increment Compared to the Previous Testing (*p*)	Correlation between VNT and ELISA (R_s_, *p*-Value)
0	0	-	0	-	-
1	4.3, 2.8–4.3 (2.3–6.3)	0.009 ^1^	45, 23–63 (17–164)	0.009 ^1^	0.91, *p* < 0.001 ^1^
2	5.8, 5.3–6.3 (4.3–8.3)	0.014 ^1^	87, 63–111 (16–146)	0.030 ^1^	0.88, *p* < 0.001 ^1^
3	6.3, 5.3–7.3 (5.3–8.3)	ns	107, 92–135 (60–174)	0.030 ^1^	0.90, *p* < 0.001 ^1^
4	6.8, 6.3–7.8 (5.3–8.3)	ns	108, 100–138 (72–173)	ns	0.79, *p* = 0.003 ^1^
5	6.8, 6.3–7.8 (5.3–8.3)	ns	114, 99–130 (70–169)	ns	0.74, *p* = 0.006 ^1^
6	6.8, 6.3–7.3 (5.3–8.3)	ns	123, 93–138 (69–169)	ns	0.84, *p* = 0.001 ^1^
7	6.8, 6.3–8.3 (5.3–9.3)	ns	122, 107–131 (79–148)	ns	0.82, *p* = 0.001 ^1^
8	6.8, 6.3–8.3 (5.3–9.3)	ns	104, 101–135 (78–168)	ns	0.91, *p* < 0.001 ^1^
9	6.3, 5.3–7.3 (5.3–8.3)	ns	136, 96–161 (68–173)	ns	0.30, *p* = 0.353
10	6.3, 5.3–6.3 (4.3–8.3)	ns	117, 97–140 (51–168)	ns	0.90, *p* < 0.001 ^1^
11	6.3, 5.3–7.3 (5.3–8.3)	ns	132, 94–137 (51–159)	ns	0.70, *p* = 0.011 ^1^
12	5.8, 5.3–6.3 (5.3–9.3)	ns	128, 113–139 (78–184)	ns	0.92, *p* < 0.001 ^1^

Antibody titers in VNT (presented as binary logarithm) and sample-to-positive control ratio (S/P%) in indirect ELISA are presented as the median, interquartile range (IQR) and range. ^1^—statistically significant at α = 0.05. ns—non-significant.

## Data Availability

The datasets used and/or analyzed during the current study are available via e-mail from the corresponding author on reasonable request.
